# Reimbursement decision-making system in Poland systematically compared to other countries

**DOI:** 10.3389/fphar.2023.1153680

**Published:** 2023-10-13

**Authors:** Aneta Mela, Elżbieta Rdzanek, Janusz Jaroszyński, Marzena Furtak-Niczyporuk, Mirosław Jabłoński, Maciej Niewada

**Affiliations:** ^1^ Department of Experimental and Clinical Pharmacology, Centre for Preclinical Research and Technology (CePT), Medical University of Warsaw, Warsaw, Poland; ^2^ Department of Administrative Proceedings, Faculty of Law and Administration, Marie Curie-Sklodowska University, Lublin, Poland; ^3^ Department of Public Health, Medical University of Lublin, Lublin, Poland; ^4^ Department of Orthopeadics and Rehabilitation, Medical University of Lublin, Lublin, Poland

**Keywords:** reimbursement decision-making systems, Polish Agency for Health Technology Assessment Tariffication, HTA agencies, systematic review, reimbursement recommendations

## Abstract

**Introduction:** Our objective was to analyze and compare systematically and structurally reimbursement systems in Poland and other countries.

**Methods:** The systems were selected based on recommendations issued by the Polish Agency for Health Technology Assessment and Tariffication (AHTAPol), which explicitly referred to other countries and agencies). Consequently, apart from Poland, the countries included in the analysis were England, Scotland, Wales, Ireland, France, Netherlands, Germany, Norway, Sweden, Canada, Australia and New Zealand. Relevant information and data were collected through a systematic search of PubMed (Medline), Embase and The Cochrane Library as well as competent authority websites and grey literature sources.

**Results and discussion:** In most of the countries, the submission of a reimbursement application is initiated by a pharmaceutical company, and only a few countries allow it before a product is approved for marketing. All of the agencies analyzed are independent and some have regulatory function of reimbursement decision making body. A key criterion differentiating the various agencies in terms of HTA is the cost-effectiveness threshold. Most of the countries have specific mechanisms to improve access to expensive specialty drugs, including cancer drugs and those used for rare diseases. Reimbursement systems often lack consistency in appreciating the same stages, leading to heterogeneous decision-making processes. The analysis of recommendations issued in different countries for the same medicinal product will allow a better understanding of the relations between the reimbursement system, HTA assessment, stakeholders involvement and decision on reimbursement of innovative drugs.

## 1 Introduction

Health care systems around the world are currently facing similar challenges of ageing populations and increased demand for medical services, intensive medical development, more and more expensive medical technologies, and an increase in public expectations of treatment options and benefits (Bittner et al., 2013). The demand for faster and more efficient access to medical services coincides with limited health care financing options. Therefore, all health systems must develop a way of prioritizing and rationing medical services in such a way that it best meets the needs of patients. In this regard, health technology assessment (HTA) enables efficient use of resources and helps to rationalize medical technology spending, as well as to set priorities (Garrido et al., 2008; Banta et al., 2009).

Countries examining the same clinical and economic evidence may make different coverage decisions (Vogler et al., 2017; Beletsi et al., 2018). Poland is an example of a country where no drug is reimbursed without formal and rigid assessment and a well-regulated process. Following the provisions of the Act of 12 May 2011 on Reimbursement of Medicinal Products, Foodstuffs Intended for Particular Nutritional Uses and Medical Devices, the manufacturer or importer of the drug must submit an appropriate application to the Ministry of Health (Barnieh et al., 2014).

In the case of a drug that has no equivalent (a new substance or a new indication), both the submission of an application and its evaluation by the AHTAPol (the Polish Agency for Health Technology Assessment and Tariffication) are required. AHTAPol is an independent organizational entity that collects data, conducts analyzes and issues reimbursement recommendations regarding the legitimacy of financing medicinal products, medical devices, foodstuffs for particular nutritional uses and health services from public funds. AHTAPol is also an advisory body for the Minister of Health—the collected analytical data and recommendations of AHTAPol support the Minister of Health in making reimbursement decisions. In the next stage, the complete file is submitted to the Economic Commission, which conducts negotiations with the applicant to determine the official selling price, the level of payment, and the indications on which the drug is to be reimbursed. Only with the recommendation of the President of the AHTAPol and with consideration to the position of the Economic Commission, taking into account the criteria listed in Article 12 of the aforementioned Act, the Minister of Health makes the final decision on a given drug for the requested indication (Husereau et al., 2014; Mihajlović et al., 2015; Kawalec et al., 2016).

The main purpose of the paper is to analyze and compare in systematical and structural way reimbursement systems in Poland and other countries.

## 2 Material and methods

Systems covered by the comparison were selected based on recommendations issued by the AHTAPol, which explicitly referred to other countries and agencies. Consequently, apart from Poland the countries included in the analysis were England, Scotland, Wales, Ireland, France, Netherlands, Germany, Norway, Sweden, Canada, Australia and New Zealand. The selection of the HTA agencies included resulted from Health Technology Assessment and Tariff Assessment Agency practice and explicit quoting in its recommendations. AHTAPol does not publish a formal list of reference agencies, thus we decided to identify them based on practice and published recommendations.

### 2.1 Research search strategy

**FIGURE 1:**
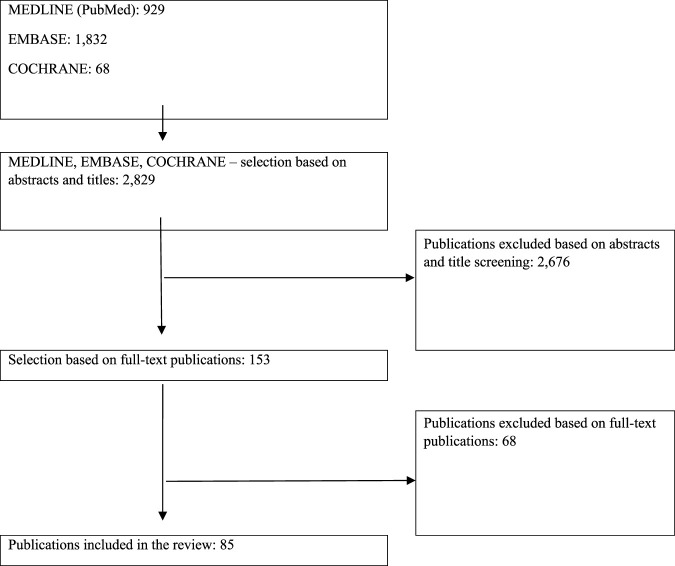
Diagram of subsequent search and selection stages (PRISMA diagram).

The bibliographic databases searched include PubMed (Medline), Embase and The Cochrane Library. The strategy for searching the databases is shown in Supplementary Appendix S1 (Supplementary Tables SA1–SA3) and was focused on HTA and reimbursement systems. No language filters were used. The last update was carried out on 22.03.2022. The search process also used references of primary reports found. Additional sources of information were the websites of individual HTA agencies and national decision-making bodies as well as publicly available grey literature. The first step in searching the databases was to identify papers that describe the drug reimbursement systems in the countries analyzed and the analytical framework described in Table 1. The selection of studies was made independently by two researchers (A.M., E.R.). As a result of the database searches, 2,829 articles and abstracts were initially evaluated for compatibility with the study’s scope. Then, after verifying the compatibility of the title and abstract with the subject of the analysis and eliminating replications, 153 papers were analyzed in detail against the inclusion and exclusion criteria. The study eventually included 85 full-text papers. Figure 1 Supplementary Appendix S1.

**TABLE 1:** Analytical framework for data extractions.

Analytical framework	Scope
The initiator of the reimbursement process	• When can the reimbursement be applied for?
	• Who submits the reimbursement application
	• To whom the application should be submitted
Health technology assessment	• HTA agencies - role and functions in systems
	• What technologies are being evaluated
	• Scientific evidences used in the HTA report
	• HTA methods and analytical techniques
Price negotiations and reimbursement decision	• Who enters price negotiations
	• Who makes reimbursement decisions
Reimbursement criteria for expensive cancer and orphan drugs	• Mechanisms used to evaluate expensive cancer drugs and those used in rare diseases
Duration of the reimbursement process	• Statutory duration of the reimbursement process
	• The actual duration of the reimbursement process

### 2.2 Data extraction and analysis

Data from the articles were extracted using a pre-designed analytical framework, the main purpose of which was to collect data on the general characteristics of the reimbursement systems in the countries analyzed.

## 3 Results

A comparison of the reimbursement systems in the countries analyzed is presented below and in Supplementary Appendix S2 (Supplementary Tables SA4–SA8).

### 3.1 The initiator of the reimbursement process

#### 3.1.1 When can reimbursement application be submitted?

In Poland and most of the countries analyzed, the availability of the product on the market is a condition for applying for reimbursement. However, it is worth noting that in some countries (e.g., Wales and Canada), an application for reimbursement can be submitted prior to marketing authorization, i.e., during the authorization procedure. Alike in England for some cancer drugs (World Health Organization, 2018).

In Canada, in the case of the Common Drug Review (CDR) program, the application covers all provinces except Quebec, where the process is conducted by INESSS (The Institut National d'Excellence en Santé et en Services Sociaux), and can be initiated by the sponsor—both before and after the issuance of the NOC (permit). In the case of pan-Canadian Oncology Drug Review (pCODR), the application can also be initiated either before or after the NOC (Notice of Compliance) is issued. Application to INESSS begins with the manufacturer’s application for registration and can be initiated at any time (except for generic drugs and natural products, for which there are relevant deadlines) (The Canadian Agency for Drugs & Technologies in Health, 2022).

#### 3.1.2 Who files the reimbursement dossier?

In Poland the reimbursement process begins with the submission of the application and relevant files to the Ministry of Health as well as processing fee payment by the market authorization holder. When applying for reimbursement for a drug that has no equivalent in a given medical indication on the reimbursement list, the Minister of Health forwards the reimbursement dossier (if it meets the formal requirements) to the Agency for Health Technology Assessment and Tarification (AHTAPol) (Dziennik Ustaw, 2011).

In England, Ireland and Wales, unlike most countries, the initiator of the reimbursement process is primarily the government. In Scotland the reimbursement process begins at the request of the marketing authorisation holder (MAH) or patients. Among the countries analyzed, the application for drug reimbursement can be submitted by various parties in healthcare - not only the responsible party, but also patients and clinicians. In majority of other countries, only pharmaceutical companies apply for reimbursement for medicinal products (Hutton et al., 2006; PHARMAC, 2017).

#### 3.1.3 To whom should the application be submitted?

In some countries, the reimbursement dossier is submitted to the Ministry of Health in Poland, Ireland and Netherlands, while in others, the dossier goes straight to the agency responsible for HTA (England, Scotland, Wales, France, Germany, Norway, Sweden, Canada, Australia and New Zealand) (Gauld, 2014; International Health Care System Profiles, 2020).

### 3.2 Health technology assessment

#### 3.2.1 HTA agencies—role and functions in systems

In the countries analyzed, HTA agencies function mainly in the form of autonomous government bodies. The duties of HTA agencies can be carried out either by regulatory bodies responsible for reimbursement decisions, or by advisory bodies that provide their reimbursement recommendations to decision-makers (such as the Ministry of Health) (Ragupathy et al., 2012; Barron et al., 2015; Wüller et al., 2015; Varnava et al., 2018).

The regulatory function is executed by agencies such as G-BA (The Federal Joint Committee, Germany), TLV (The Dental and Pharmaceutical Benefits Agency, Sweden), NoMA (The Norwegian Medicines Agency, Norway), PHARMAC (The Pharmaceutical Management Agency, New Zealand), NICE (The National Institute for Health and Care Excellence, England) and SMC (The Scottish Medicines Consortium, Scotland). If the recommendation is negative, price negotiations can take place where the MAH can further demonstrate the cost-effectiveness of the therapy by lowering the price (Schwarzer and Siebert, 2009; Bending et al., 2012; Weise, 2018). In some countries, there are more institutions that have different functions and undertake HTA activities at the national level (Ireland, Germany, Sweden, Norway and Canada) (CADTH, 2021; Fontrier et al., 2022).

In countries where agencies have an advisory role, positive reimbursement recommendations do not always translate into final reimbursement coverage of a technology; also their impact on the outcome of price negotiations is not clear (Wales, Ireland, Netherlands, Canada). The recommendation of the President of AHTAPol is also not conclusive, as the decision of the Minister of Health is mainly influenced by arrangements occurring at later stages of the process, such as a reduction in the drug price by MAH during negotiations. In Poland the outcome of the AHTAPol assessment may have a significant impact on the price negotiations (Fontrier et al., 2022).

Different systems have adopted different organizational arrangements of reimbursement process, and the HTA agencies organization may also very. Most countries have a “light” model, under which agencies assess the quality of the analyses (including their reliability, and objectivity) (Xie et al., 2011). Other countries have a “heavy” model—the agencies develop the analysis internally. Such organizations require substantial public funding (British agency NICE, Swedish agency SBU). NICE’s HTA process takes into account both cost-effectiveness analyses that have been developed by external centers on behalf of NICE and analyses submitted by the manufacturer. The two types of documentation have different impacts on decision-making, but the use of both allows for full aggregation of data and provides clear benefits to the healthcare system. It is understood that the Polish AHTAPol operates on a mixed model—the agency conducts its own analyses and is responsible for data retrieval, but also assesses the reliability of the analyses submitted by the applicant. HTA evaluation guidelines are available in all countries analyzed (Vreman et al., 2020; Fontrier et al., 2022).

#### 3.2.2 Scientific evidence reported in the HTA

The countries analyzed can be categorized based on whether they consider as part of their HTA evaluation only clinical effectiveness and safety or both clinical benefit and cost-effectiveness, including the impact on the payer’s budget for health services (Lloyd et al., 2009; Gulácsi et al., 2012; Grigore et al., 2020).

Poland, England, Scotland, Wales, Netherlands, Germany, Norway, Sweden, Canada, Australia and New Zealand, consider both the clinical and economic aspects, including impact on the payer’s budget for health services. In addition, in some countries additional aspects are evaluated, such as unmet medical need, the degree of innovation of the therapy being evaluated, and ethical, social, legal and organizational aspects (Oortwijn et al., 2002; DeJean et al., 2009; Rowen et al., 2017; Agencja Oceny Technologii Medycznych i Taryfikacji, 2022; Schurer et al., 2022; The Dental and Pharmaceutical Benefits Agency, 2023).

In most countries single technology are usually evaluated. England and Norway have three formats for developing the report. In England, HTA can be conducted as part of a multiple technology assessment—MTA (this is an assessment of several drugs or therapies used in one indication or one technology in several indications), as part of a single technology assessment—STA (technology assessment of one drug or therapy in one indication), or as part of fast technology assessment - FTA (this is used to provide patients with faster access to the most cost-effective new treatments). In Norway, in addition to STA and MTA, a so-called mini HTA is used—a simplified assessment performed by clinical experts for in-hospital technologies (Gulácsi et al., 2012; CADTH, 2021).

#### 3.2.3 Data source, HTA methods and analytical techniques

All countries recognize a variety of data sources, including scientific studies (clinical trials, observational studies), national statistics, clinical guidelines, surveys, expert reports and other evidence from pharmaceutical manufacturers (Ringborg et al., 2003; Marshall et al., 2008; Novaes and Soárez, 2016).

While some countries use costs to assess the value of a drug, others, such as France, evaluate the relative clinical benefit of the drug compared to existing comparators and use evidence from randomized clinical trials. The relative benefit of a drug is assessed on a 5-point scale based on the level of improvement due to clinical benefit (ASMR - Amélioration du Service Médical Rendu), which is used to determine the reimbursement level (Haute Autorité de santé, 2023a). HTA agencies vary in their willingness to accept indirect comparisons and have individual priority regarding the methodology used for comparison of indirect treatments. Only The Federal Joint Committee (G-BA) emphasizes the need for direct evidence and a requirement for convincing arguments to justify not having head-to-head data (Es-Skali and Spoors, 2018). The assessment of cost-effectiveness of drugs varies across countries, with each country having its own set of guidelines and thresholds -Supplementary Appendix S2 (Supplementary Table SA7) (Swedish Agency For Health Technology Assessment And Assessment Of Social Services, 2007; Corbacho and Pinto-Prades, 2012; Kolasa and Wasiak, 2012; Ragupathy et al., 2012; Streat and Munn, 2012; Zyśk and Niewada, 2013; Chabot and Rocchi, 2014; McCullagh and Barry, 2016; DentalPharmaceutical Benefits Agency, 2017; Jahnz-Różyk et al., 2017; Schaefer et al., 2021; National Health Care Institute, 2022; The Pharmaceutical Management Agency, 2023)^.^

### 3.3 Price negotiations and reimbursement decision

Negotiations play a crucial role in reimbursement decisions in most of the countries analyzed, for both original and generic drugs.

In Poland, an important step on the road to reimbursement is negotiation conducted by the Economic Commission appointed by Ministry of Health. The scope of these negotiations includes *inter alia* setting the official selling price in Poland, clinical indication under which the drug is to be reimbursed, and managed entry agreements (MEA) (Jahnz-Różyk et al., 2017). According to the Reimbursement Act, there are five types of MEA designed to ensure the availability of innovative drugs within the budget constraints of the public payer. These MEA types are as follows: a) pay for effect–reimbursement is contingent upon the drug’s demonstrated effectiveness in achieving desired outcomes; b) price discount–the drug manufacturer and payer agree upon a discount for the drug’s price; c) price volume–involves determining the number of drug packages or the size of the population for which the drug will be sold at a prearranged price; d) pay-back–the drug manufacturer is obligated to return part of the reimbursement received from public funds, e.g., for each reimbursed packaging or after exceeding the predetermined amount that was intended to finance a given active substance; e) other–this category encompasses additional reimbursement conditions aimed at increasing the availability of guaranteed benefits or reducing associated costs. These MEA types collectively contribute to ensuring that patients have access to innovative drugs, even within the limitations of the public payer’s budget. They establish a framework that incentivizes the effectiveness, affordability, and appropriate use of these medications for the benefit of patients and the healthcare system (Dziennik Ustaw, 2011). The findings of the analysis of the risk-sharing instruments proposed in the reimbursement applications (for drugs used in oncological diseases under drug programs assessed by the Polish HTA Agency in 2012–2018) highlight a predominant preference for easy-to-implement mechanisms, with rebates or pay-back emerging as the dominant choices. These mechanisms were favored for their simplicity and user-friendly nature, allowing for seamless integration into the existing reimbursement framework (Iwańczuk et al., 2018).

In Netherlands, as in Poland, price negotiations with a pharmaceutical company influence the type of decision the Ministry of Health, Welfare and Sports makes. According to the Drug Pricing Law, the ministry sets maximum drug prices, which are subject to adjustment every 6 months—the reference point being drug prices in reference countries (Belgium, Germany, France and the United Kingdom) (Sharaf, 2014; Goverment of the Netherlands, 2023).

In Norway, The Norwegian Drug Procurement Cooperation (LIS) is responsible for negotiating prices for reimbursed drugs. If a positive reimbursement decision involves an increase in reimbursement expenditures of 10 million Euro over 5 years, then the NoMa is not authorized to grant reimbursement. In this case, as long as the application meets all the prioritization criteria, NoMA forwards the assessment to the Ministry of Health and Welfare (Directorate For Financial and Enterprise Affairs, 2014; Report, 2020).

In Sweden, the institution responsible for reimbursement decisions and pricing is the Dental and Pharmaceutical Benefits Agency (TLV). Decisions on reimbursement and pricing of new drugs are made by an expert committee within the agency, i.e., The Pharmaceutical Benefits Board, which is appointed by the government and consists of seven members recruited from district councils, universities or patient organizations. The agency’s decisions are binding at both the national and local levels (district councils). The degree and implementation rate may vary from district to district due to individual budgeting mechanisms or different interpretations of the Agency’s recommendations (Carlsson et al., 2000).

In some countries negotiations are conducted only if cost-effectiveness is not demonstrated in the HTA. In Ireland, if a drug is cost-effective, the Health Service Executive (HSE) may approve the drug for reimbursement. Otherwise, the drug is referred for evaluation by the HSE Drugs Group. At this stage, it is possible to conduct commercial negotiations with the applicant to work out mutually acceptable financing terms, or to enter into patient access schemes, which are reimbursement individual decisions (Global Legal Insights, 2020a; Government of Ireland, 2020).

In England, any product that NICE has assessed as cost-effective, but may cost the NHS more than 20 million GBP in any of its first 3 years of use, must be subject to further negotiation between the manufacturer and the NHS. If negotiations fail, the NHS can ask NICE to delay funding for the product for up to 3 years (or longer—in exceptional cases). The Secretary of State for Health and Welfare is responsible for negotiating prices with pharmaceutical companies under the Pharmaceutical Price Regulation Scheme (PPRS), which is a voluntary agreement between the Department of Health and the Association of the British Pharmaceutical Industry (ABPI) for the supply of licensed, brand-name drugs to the NHS (Global Legal Insights, 2020b). In Scotland, in the case of a positive decision and approval of a drug for use by NHS Scotland, each of the 14 local health boards makes an individual decision based on the SMC’s recommendation. Area Drug and Therapeutics Committees (ADTCs) ultimately decide on public funding for a drug in a given area. Typically, local NHS decisions are consistent with those of the SMC, thus avoiding regional variation in prescribing. The main differentiating element between the SMC and the ADTC is that the former authority considers the drug’s effect/price ratio, while regional institutions focus on affordability (Scottish Medicines Consortium, 2023). Drugs that are ineligible for evaluation by national bodies (AWMSG—Wales, NICE—England) may be included in the list of NHS-funded drugs as a result of a decision by a local health board or NHS Trust decision-making group. Each of these institutions has its own drug introduction process, which evaluates the available evidence on efficacy, safety, cost-effectiveness and budget impact. The decision determines the drug’s place in therapy (e.g., line of treatment) and who can prescribe the drug (All Wales Therapeutics and Toxicology Centre, 2023).

In other countries (i.e., France and Germany) the outcome of the clinical assessment is a key factor in price negotiations. In Germany if additional benefits are proven, within 6 months the GKV-SV (The National Association of Statutory Health Insurance Funds) and the pharmaceutical company negotiate the reimbursement price (The Federal Joint Committee, 2023). In France, once pharmaceuticals or medical devices are approved for reimbursement based on a favorable opinion from the relevant HAS committees, their prices are negotiated with the CEPS (Economic Committee for Health Products). The final reimbursement decision driven by the price and total reimbursement spending, is issued by the relevant ministry and published in the Journal of Laws of the French Republic within 180 days of the application submission (Haute Autorité de santé, 2023b).

In Canada, after CADTH (or INESSS - in the case of Quebec) issues a positive recommendation, the pan-Canadian Pharmaceutical Alliance (pCPA) starts negotiations. In the first instance, the provinces decide whether negotiations will take place. They can either forgo negotiating a price or decide to negotiate collectively or individually. Provinces can also negotiate for the CADTH’s non-evaluated drug. If a decision is made to negotiate, a jurisdiction is chosen to represent all provinces in the negotiations. Negotiations are conducted with the participation of the provincial representative and the manufacturer and may include other terms in addition to the price (Dziennik Ustaw, 2011). If an agreement is reached, the manufacturer and the provincial representative sign a Letter of Intent (LOI). Based on that, the terms of the Product Listing Agreement (PLA) with the manufacturer are negotiated for the relevant provinces. Provinces can opt out of PLA negotiations. It should be noted that CADTH recommendations are not binding, and each region makes its own reimbursement decisions based on CADTH recommendations and other factors, such as financial constraints. In the case of INESSS, the final decision is made by the Minister of Health, and it is usually consistent with the INESSS recommendation. CDR’s recommendations are not binding, and decisions for the province are made separately (Dziennik Ustaw, 2011).

In Australia, although the final decision is made by the Minister of Health, it cannot be other than the PBAC’s recommendation. In cases where a decision to place a drug on the PBS list would result in a net cost exceeding USD 20 mln per year, the decision is made by the Federal Cabinet prior to the Minister’s decision (Pharmaceutical Benefits Advisory Committee, 2023).

In New Zealand, PHARMAC negotiates prices for inpatient, outpatient and cancer treatment, as well as prices for pharmaceuticals, vaccines and medical devices, and manages the limited state budget for outpatient and cancer drugs67^,^68.

### 3.4 Specific approach for cancer and rare-disease drugs

Some countries have established mechanisms to improve access to highly specialized drugs, including those used to treat cancer and rare diseases (Simoens, 2011; Pauwels et al., 2014; Adkins et al., 2017; Stawowczyk et al., 2019; Zamora et al., 2019).

Poland has what is known as rescue access to drug technology (RADT), a system for issuing individual approvals for the treatment of patients for whom all publicly funded therapeutic options have been exhausted. Medicines financed under RADT are free of charge to patients, and their cost are covered by the NHF. Only the healthcare center directly involved in patient treatment can submit an application for RADT funding. Funding may cover drugs whose appropriateness for use in a particular case has been confirmed by a national or provincial consultant. However, the decision is issued for a specified period of time, after which the effectiveness of the treatment and the legitimacy of its continuation are assessed. An alternative path to access to drugs with high clinical innovation is financing under the Medical Fund (Ustawa z dnia 27 sierpnia 2004r, 2004).

In England, the HST process is available for products that are dedicated to clinically distinct target patient group, which treatment is concentrated in a small number of NHS centers, the patient’s condition is chronic and severely disabling, and the technology has the potential for lifelong use. For these products, conventional NICE assessment assumes some allowance to account for the likely higher costs and often more limited clinical data. NICE typically recommends HTS which ICER is less than 100,000 GBP. In certain circumstances, based on clinical benefit magnitude, NICE can recommend products above this threshold, usually up to 300,000 GBP (Ragupathy et al., 2015). England also has a Cancer Drugs Fund (CDF), established in 2011. Its rules were changed in July 2016 to facilitate faster access to promising new treatments. Further evidence is also collected to eliminate clinical uncertainty (Ragupathy et al., 2015).

In Scotland, the SMC uses different evaluation criteria (MCDA, multi-criteria decision analysis) and places less emphasis on the cost-effectiveness of cancer and rare diseases drugs. More weight is given to effects that are not necessarily quantified (e.g., the impact on the patient’s family or ability to work). If the drug meets the standard ICER criteria, the SMC may temporarily approve it. If not, the Peer and Clinician Engagement (PACE) process is initiated, which is not used in the standard approach. The PACE process relies on a consultative approach between patients and clinicians, who, identify additional effects that are relevant from the perspective of the patients, but difficult to quantify with ICER. This approach makes it possible to allow more expensive drugs to be used in the NHS. In the event of a negative SMC recommendation for a drug, a patient can access it using either tier 1 or tier 2 PACS, with tier 1 covering ultra-rare diseases and tier 2 covering all drugs that have not been approved by the SMC (Ragupathy et al., 2015).

In France, as an exception, innovative or very expensive drugs may be financed under an additional “reimbursement tariff” determined in advance by specific agreement with CEPS (Economic Committee for Health Products) (Weise, 2018).

As in England and Scotland, higher values for cost-effectiveness are accepted in Canada for rare diseases and cancer drugs. For this purpose, a special team was set up to evaluate and make relevant recommendations, the pCODR, which replaced the Joint Oncology Drug Review (JODR) as of 2010. The evaluation process conducted by pCODR is formalized and time-framed, and takes into account the opinions of clinicians, economic experts, manufacturers and patients. The process of evaluating cancer drugs is similar to that used in treatments for other diseases and is based on analogous premises and data (Janoudi et al., 2016; Nigel, 2018).

In Germany, Australia and New Zealand, end-of-life medical technologies are not evaluated under a different procedure. In Germany, the legislator specifies that the additional medical benefit has already been proven in the registration process. Therefore, the assessment for the additional benefit categories are omitted for orphan drugs. Furthermore, for orphan drugs, an additional benefit does not have to be proven by comparison to an appropriate comparative therapy that had been previously approved by the G-BA77.

Australia has another way of funding very expensive and cost-ineffective treatments for rare diseases, so called The Life Saving Drugs Program. It applies only to treatments which reduce mortality and prolong patient survival. There are no additional restrictions on the diseases or treatments that can be covered by the program. Reimbursement is on a patient-by-patient basis and depends on 10 criteria (including efficacy and expected impact on patient survival, lack of alternative treatment and significant disease-related mortality, the high price of the technology, which is a significant limitation on the patient’s personal funding) (Government of Ireland, 2020).

In New Zealand, the Rare Disorders Advisory Committee may reconsider the funding application if new evidence is provided (International Health Care System Profiles, 2020).

In Wales, Ireland and Norway there are currently no distinct financed pathway for oncological and orphan drugs (Janoudi et al., 2016; Schulz et al., 2020).

### 3.5 Time to reimbursement

The regulatory time limit for the reimbursement process varies among countries. In majority of the countries the total duration of the process, from submission of an application to issuance of a reimbursement decision, should take no longer than 180 days. Norway and Canada have adopted a time limit of 90 days (for non-cancer drugs), while the process takes the longest in England, up to 305 days. However, it should be noted that in practice, decisions are rarely made within the timeframe specified in the regulations. Independently conducted analyses indicated the time to drug reimbursement is the longest in Poland (844 days), followed by New Zealand (789 days), Canada (602 days), Ireland (541 days), France (497 days), Australia (467 days), Scotland (417 days), Norway (414 days), England (340 days), Netherlands (294 days), Sweden (261) and Germany (133 days) (IQVIA, 2021; EFPIA Patients, 2022).

Considering the aspects analyzed within each country, the following reasons for the limited availability of innovative therapies can be identified.

Although in some countries (England, Canada) it is possible to apply for reimbursement while still in the registration process this does not always translate into faster access to new drugs. An example of such a country is Canada. The reasons for the long delays in the introduction of new drugs on the market include the multi-layered sequential review process conducted before public drug plans make new innovative therapies available (Salek et al., 2019).

In countries where reimbursement recommendations published by individual HTA agencies are binding, i.e., a positive recommendation equals reimbursement coverage (England, Scotland, Germany, Norway, Sweden), the time from registration to reimbursement is much shorter. The exception is New Zealand, where despite the regulatory function of the HTA agency, the actual average duration of the reimbursement process is time consuming. Finally, the implementation of the HTA recommendations can also vary, depending on the advisory or regulatory role of the HTA agency. In the case of Sweden, where the national HTA agency is a regulatory body which decisions are adopted by local administrative councils, not all councils can afford all reimbursement decisions, which can lead to inter-regional differences.

The countries analyzed also have different approaches to in hospital drugs, generic and innovative drugs, the length of time for which a reimbursement decision is issued, and how drug expenses are accounted for and whether drugs should be replaced with cheaper substitutes. Finally negotiations may contribute substantially to longer time to reimbursement decision.

The presence of specific mechanisms or more lenient criteria in the evaluation of clinical and economic evidence for cancer and rare disease drugs is also an important factor affecting access to breakthrough therapies. Poland is an example of a country where less restrictive HTA evaluation criteria do not apply. Poland has no specific reimbursement arrangements on drugs in rare diseases, which could be perceived as systematic approach to facilitate accurate and timely patients access. Medical Fund regulation only partially addresses that challenge for highly innovative products only.

## 4 Discussion

This paper addresses the similarities and differences of the reimbursement systems (with particular emphasis on HTA assessment) in Poland and 12 countries, which HTA bodies’ recommendations are most often quoted by the AHTAPol. In other similar studies usually either a smaller number of countries or a narrower range of aspects were covered (Barnieh et al., 2014). Individual publications covered more countries, but their scope of analysis focused on selected stages or aspects of the reimbursement process (Fontrier et al., 2022). The aim of Fontrier et al. systematic review was to examine system design and its impact on the financing of technologies that are subject to HTA evaluation. It focused on 32 countries, including countries in the European Union, Australia, Canada and the United Kingdom, presenting a comparison of the key operational functions of the HTA systems (Fontrier et al., 2022). Another study concentrated on the processes in 35 reimbursement systems in 33 OECD countries where pharmacological drugs are publicly financed (Barnieh et al., 2014). However, the authors focused solely on the comparison of the initiators of the reimbursement process and the requirement for clinical evidence. In addition, they conducted an assessment of the fairness of each reimbursement system based on three criteria (transparency of decisions, consideration of clinical and economic evidence, and appealability) (Barnieh et al., 2014). Our systematic review evaluates all key stages of the reimbursement process including among others: the HTA agencies policy and criteria used in HTA assessment as well as price negotiations related aspects and specific approach for expensive innovative medicinal products. Despite covering a smaller number of countries (which is a direct result of the credentials of the Polish HTA agency) it is, to the best of our knowledge, the first such comprehensive analysis.

In most of the countries that have been studied, the process of submitting a reimbursement application is initiated by the pharmaceutical company. Only a few countries allow submission before a product is approved for marketing. The next steps in the reimbursement process are similar across most countries. After a formal and legal evaluation of the application, it is sent for assessment by the HTA agency/authority. In several countries the assessment, appraisal, and subsequent final recommendation have a binding effect on the drug’s coverage from the public budget. Price negotiations take place in each country, although in some cases, it is not mandatory, such as when ICER (incremental cost-effectiveness ratio) is below the threshold. Regarding subsequent stages of the reimbursement process, such as the reimbursement decision and the institution responsible for financing the reimbursed drug, there are significant differences from country to country. Although the reimbursement systems share the same goal, the way to final decision and HTA use to support it can be organized much differently, thus one universal model cannot be claimed, and studies of existing arrangements are justified.

More similarities can be seen in the functioning of HTA agencies and their role in the reimbursement system. All countries covered have an agency responsible for HTA evaluation, with only a few exclusively evaluating pharmaceuticals. Historically in many countries, Poland is a model example, initial role of the agency evolved and more responsibilities were added. National evaluations are conducted by all agencies, with some also performing regional assessments. The agencies are independent and some also serve as regulatory bodies for reimbursement decision making. The HTA assessment criteria reviewed in most countries include unmet medical needs, degree of innovation, clinical effectiveness, and safety profile. Most agencies also consider cost-effectiveness and impact on the payer’s budget. Only three countries explicitly include ethical, social, legal, and organizational aspects in their evaluation, indicating a lack of emphasis on these criteria by most HTA agencies. The methods and analytical techniques used in HTA are generally consistent across all agencies reviewed. The cost-effectiveness threshold is a key differentiator between agencies, with nearly half of the countries not having an officially established value for cost-effectiveness.

Established in 2005, the EUnetHTA initiative, which mission is to foster cooperation among European institutions involved in HTA assessment, confirms the convergence of the various HTA agencies’ approaches on the issue of health technology assessment, especially clinical evaluation. The cooperation is expected to be beneficial from a European as well as a national and regional perspective, with the overarching goal of improving access to therapies (Wang et al., 2020; Julian et al., 2022). An approach towards common clinical assessment shared by many countries may significantly reduce the burden on individual countries and should have a positive impact on reducing the time to reimbursement decision.

Some authors point out that although countries implement HTA, the way in which this assessment fits into the reimbursement process (at the negotiation, decision-making, coverage determination, and funding stages) differs significantly from one system to another (Fontrier et al., 2022). The review by Fontrier et al. covering also the countries not included in our study showed a different and inconsistent approach to valuing clinical evidence and outcomes reported in clinical trials (Fontrier et al., 2022). The similarities of different reimbursement systems in terms of HTA may result from adaptation by countries the standards of countries more experienced. Authors of another study observed a convergence in the criteria considered in HTA assessments by all countries included in the analysis, while at the same time observing differences in the approach of countries where HTA was introduced earlier from those where the assessment was introduced relatively recently (after 2000) (Barnieh et al., 2014). The first group of countries places significant emphasis on improving the quality of care, ensuring equal access to treatment and efficient use of resources. The second group, on the other hand, focuses primarily on the aspect of the impact of introducing new therapies on the payer’s budget. Countries in this group, despite having their own official guidelines, often follow the decisions of other reimbursement systems more experienced in HTA evaluation.

Reimbursement systems were initially introduced to enhance patients’ access to effective therapies. However, these systems often lack consistency in how they organize and appreciate the various stages involved. As a result, the decision-making process can become heterogeneous and less transparent.

While many systems utilize HTA to inform reimbursement decisions, the implementation of HTA itself is not uniform. The inconsistency may stem from the nature of the final decision-making process, which can either be a logical, step-by-step, multi-stakeholder-driven, integrated, and longitudinal process or a decision made independently by the responsible body (usually the Ministry of Health, as in the case of Poland), based on recommendations or statements from other auxiliary bodies.

Undoubtedly, these different arrangements can lead to variations in the time it takes to reach a decision and the genuine access patients have to drugs. It should be noted, however, that the time that elapses between registration and the availability of a drug to patients is long not only due to delays caused by the institutions involved in the reimbursement process but may also be the result of the strategy adopted by the pharmaceutical company, which begins the process not necessarily at the same time in each country, not always immediately after obtaining marketing authorization.

## 5 Limitations of the analysis

1) The main limitation is that only 13 countries were included in the analysis, but this is determined by Polish HTA agency practice on referring to selected agencies. As AHTAPol does not refer to agencies in CEE countries we did not include them, it can be recognized as excused approach, but certainly, CEE countries could serve as more relevant in the context of available resources and shared challenges. 2) Only publicly available data were used in the publication, making it impossible in some cases to find detailed information about the reimbursement process or the HTA agency and its health technology assessment process. 3) Dynamic settings and evolution of the systems make it difficult to study and provide no time-sensitive conclusions. To prevent this, we also verified the information available on the websites of the various HTA agencies. 4) Due to the use of published data only, it is possible that the correlation between the various aspects of HTA and their impact on the final drug reimbursement decision are not fully captured. 5) It should be noted that the real access to drugs is not solely determined by the reimbursement decision, as it serves as limited proxy and falls outside the scope of this paper.

## Data Availability

The original contributions presented in the study are included in the article/Supplementary Material, further inquiries can be directed to the corresponding author.
