# Supplementary material for: Reimbursement decision-making system in Poland systematically compared to other countries
Source: Front Pharmacol. 2023 Oct 13;14:1153680. doi: 10.3389/fphar.2023.1153680 (PMC10611478; doi:10.3389/fphar.2023.1153680)
Supplement: Supplementary file 1 [file DataSheet1.docx]

**SYSTEMATIC REVIEW**

# **Reimbursement decision-making system in Poland systematically compared to other countries**

# **Appendix 1**

# **Tab. 1. Publication search strategy in MEDLINE database system (PubMED); data as of March 14, 2022**

| **ID** | **Keywords** | **Number of results** |
| --- | --- | --- |
| #1 | "reimbursement mechanisms"[MeSH Terms] | 36 652 |
| #2 | "system"[Title/Abstract] OR "systems"[Title/Abstract] OR "mechanism"[Title/Abstract] OR "mechanisms"[Title/Abstract] OR "policy"[Title/Abstract] | 4 888 112 |
| #3 | #1 AND #2 | 9 633 |
| #4 | "technology assessment, biomedical"[MeSH Terms] | 11 130 |
| #5 | "health technology assessment"[Title/Abstract] | 4 834 |
| #6 | #4 OR #5 | 14 146 |
| #7 | "agencies"[Title/Abstract] OR "units"[Title/Abstract] OR "departments"[Title/Abstract] | 380 521 |
| #8 | #6 AND #7 | 1 065 |
| #9 | #3 OR #8 | 10 688 |
| #10 | #9 AND (Review OR Systematic Review) | 1 037 |
| #11 | **#10 AND (English OR Polish)** | **929** |

# **Tab. 2. Publication search strategy in the EMBASE database system; data as of March 14, 2022.**

| **ID** | **Keywords** | **Number of results** |
| --- | --- | --- |
| #1 | 'reimbursement'/exp | 56 805 |
| #2 | 'system':ab,ti OR 'systems':ab,ti OR 'mechanism':ab,ti OR 'mechanisms':ab,ti OR 'policy':ab,ti | 5 913 630 |
| #3 | #1 AND #2 | 14 090 |
| #4 | 'biomedical technology assessment'/exp | 14 681 |
| #5 | ‘health technology assessment’:ab,ti | 5 988 |
| #6 | #4 OR #5 | 17 932 |
| #7 | ‘agencies’:ab,ti OR ‘units’:ab,ti OR ‘departments’:ab,ti | 492 912 |
| #8 | #6 AND #7 | 1 483 |
| #9 | #3 OR #8 | 15 478 |
| #10 | #9 AND [embase]/lim | 12 336 |
| #11 | #10 AND [review]/lim | 2 001 |
| **#12** | **#11 AND ([english]/lim OR [polish]/lim)** | **1 832** |

# **Tab. 3. Publication search strategy in the Cochrane Library system; data as of March 14, 2022.**

| **ID** | **Keywords** | **Number of results** |
| --- | --- | --- |
| #1 | MeSH descriptor: [Reimbursement Mechanisms] explode all trees | 250 |
| #2 | system:ti,ab,kw OR systems:ti,ab,kw OR mechanism:ti,ab,kw OR mechanisms:ti,ab,kw OR policy:ti,ab,kw | 210 550 |
| #3 | #1 AND #2 | 114 |
| #4 | MeSH descriptor: [Technology Assessment, Biomedical] explode all trees | 143 |
| #5 | health technology assessment:ti,ab,kw | 4 158 |
| #6 | #4 OR #5 | 4 198 |
| #7 | agencies:ti,ab,kw OR units:ti,ab,kw OR departments:ti,ab,kw | 32 627 |
| #8 | #6 AND #7 | 364 |
| #9 | #3 OR #8 | 477 |
| **#10** | **#9 in Cochrane Reviews** | **68** |
| #11 | #9 in Trials | 409 |

# **Fig 1. Diagram of subsequent search and selection stages (PRISMA diagram)**

| MEDLINE (PubMed): 929  EMBASE: 1832  COCHRANE: 68 |  | | | |
| --- | --- | --- | --- | --- |
|  |  |  |  |  |
| MEDLINE, EMBASE, COCHRANE – selection based on abstracts and titles: 2,829 |  | |  |  |
|  |  | Publications excluded based on abstracts and title screening: 2,676 | |  |
| Selection based on full-text publications: 153 |  |  | |  |
|  | | | |  |
|  | | Publications excluded based on full-text publications: 68 | |  |
| Publications included in the review: 85 |  |  | |  |

# **Appendix 2**

# **Tab 4. Stages and relevant responsible bodies involved in the reimbursement process**

| **CATEGORY** | **Poland** | **England** | **Scotland** | **Wales** | **Ireland** | **France** | **the Netherlands** | **Germany** | **Norway** | **Sweden** | **Canada** | **Australia** | **New Zealand** |
| --- | --- | --- | --- | --- | --- | --- | --- | --- | --- | --- | --- | --- | --- |
| **DOSSIER SUBMISSION** | Manufacturer | Manufacturer at the request of the Department of Health and Human Services  (application may be submitted prior to authorization in the case of oncology drugs) | Manufacturer/ Patients | Manufacturer (the application may be submitted before the authorization) | Manufacturer | Manufacturer | Manufacturer | Manufacturer | Manufacturer | Manufacturer | Manufacturer (the application may be submitted before the authorization) | Manufacturer | Manufacturer / others |
| **DOSSIER EVALUATION**  **(formal and legal assessment)** | Ministry of Health  (14-30 days) | Appraisal Committee by NICE | SMC | AWTTC, NMG by AWMSG | HSE | Transparency Council by HAS | Ministry of Health, Social Welfare and Sports | G-BA | NoMA | TLV  The Pharmaceutical Benefits Board | CADTH (CDEC)  INESS | PBAC | PHARMAC |
| **REIMBURSEMENT RECOMMENDATION** | AHTAPol  (60-80 days) | NICE – ACD, FAD  (245-305 days) | SMC | AWMSG | NCPE | HAS  (90 days) | National Institute of Health (ZIN)  Scientific Advisory Board  Appraisal Committee | IQWiG  G-BA | NoMA | TLV – reimbursement decision with justification | CADTH  - CDR for non-ocological drugs  - pCODR for oncological drugs  INESS for Quebec  - CSMEI for all medicinal products | PBAC | PHARMAC  PTAC |
| **Does the type of recommendation affect the reimbursement decision?** | Non-binding | Binding | Binding | Non-binding | Non-binding | Non-binding | Non-binding | IQWIG – Non-binding  G-BA - Binding | Binding | SBU – Non-binding  TLV – Binding | Non-binding | Binding | Binding |
| **PRICE NEGOTIATIONS** | Economic Commission  Minister of Health | The Secretary of State for Health and Social Care  NHS England commercial | Local NHS Health Committees (ADTCs) | AWMSG | HSE - when ICER exceeds the efficiency threshold | CEPS  (90 days) | Ministry of Health, Social Welfare and Sports | GKV-SV (The National Association of Statutory Health Insurance Funds) | LIS (The Norwegian Drug Procurement Cooperation) | TLV  The country councils | pan-Canadian Pharmaceutical Alliance (pCPA) | Ministry of Health | PHARMAC |
| **REIMBURSEMENT DECISION** | Ministry of Health | NICE | Local NHS Health Committees (ADTCs) | AWMSG | HSE | Ministerstwo Zdrowia | Ministry of Health, Social Welfare and Sports | G-BA | NoMA | TLV  The Pharmaceutical Benefits Board | Teritorial drug  programs for CADTH  MoH for INESSS | Ministry of Health | PHARMAC |
| **Reimbursement list update** | Every 2 months | Every 1 month | Every 1 month | Every 1 month | Every 1 month | Every 1 month | No data | No data | Every 1 month | No data | No data | Every 1 month | Every 3 months |
| **Duration of reimbursement decision** | 2, 3 years | 5 years | No data | No data | 1 year | 5 years | 4 years | 2 years | No data | No data | No data | No data | No data |
| **DRUG REIMBURSEMENT BUDGET HOLDER** | National Health Fund  (Social Health Insurance) | NHS  (1 - 3 months after the recommendation)Regional funding | NHS  Regional funding | NHS  Regional funding | NHS  Hospitals via  own budget/  NHS for  medicines  covered under  national drug  management  programmes | SHI  Social Security within French Health Insurance National Program | Health insurers | SHI Social Health Insurance | National  insurance  scheme (SHI) – primary health care drugs  Regional  health  authorities  (hospitals) – specialized drugs | County  councils  (regions) | Provinces | Federal government | Ministry of Health / Goverment |
| **Evaluation criteria for rare-disease drugs** | Rescue Access to Drug Technology (RADT) | Specific pathway for Highly Specialized Technologies (HST)  Less emphasis on the cost-effectiveness  Cancer Drug Fund (CDF) | Peer and Clinician Engagement process (PACE)  Multi-criteria decision analysis (MCDA)  Less emphasis on the cost-effectiveness | n/a | n/a | Additional reimbursement tariff | n/a | No additional benefit should be proven | n/a | Less emphasis on the cost-effectiveness | n/a | The Life Saving Drugs Program | The application may be reassessed if new evidence will be provided |

Source: own elaboration based on systematic review

# **Tab. 5. HTA agencies - functions and empowerment in the system**

| **Category** | **Poland** | **England** | **Scotland** | **Wales** | **Ireland** | **France** | **the Netherlands** | **Germany** | **Norway** | **Sweden** | **Canada** | **Australia** | **New Zealand** |
| --- | --- | --- | --- | --- | --- | --- | --- | --- | --- | --- | --- | --- | --- |
| **HTA agency** | AHTAPol | NICE | SMC | AWMSG | NCPE  HIQA | HAS | ZiN | IQWIG  G-BA | NoMA  FHI (vaccines) | TLV (2002)  SBU | CADTH  INESS  CED | PBAC | PHARMAC |
| **Expert Comitte** | Transparency Council | Appraisal Committee | n/a | All Wales Therapeutics and Toxicology Centre (AWTTC) | n/a | The Economic and Public Health Committee (CEESP) | Scientific Advisory Board  Appraisal Committee | n/a | n/a | The Board for Pharmaceutical Benefits | n/a | Drug Utilisation Sub Committee  Economics Sub Committee | Pharmacology and Therapeutics Advisory Committee (PTAC) |
| **HTA coverage** | National | National  Regional | National | National | National | National | National | National | National  Regional | National  Regional | National  Regional | National | National |
| **Role of HTA agencies** | Advisory | Advisory  **Regulatory** | Advisory  **Regulatory** | Advisory | Advisory | Advisory | Advisory | Advisory (IQWIG)  **Regulatory  (G-BA)** | Advisory  **Regulatory** | Advisory (SBU)  **Regulatory (TLV)** | Advisory | Advisory | Advisory  **Regulatory** |
| **Medical technologies in scope** | Pharmaceuticals, medical devices and  other technologies | Pharmaceuticals, medical devices and  other technologies | Pharmaceuticals, medical devices | Pharmaceuticals | Pharmaceuticals, medical devices | Pharmaceuticals, medical devices, hospital  medical technologies | Pharmaceuticals, medical devices and  other technologies | Pharmaceuticals | Pharmaceuticals, medical devices and  other technologies | Pharmaceuticals, medical devices and  other technologies | Pharmaceuticals, medical devices and  other technologies | Pharmaceuticals | Pharmaceuticals, medical devices and  other technologies |
| **HTA agency model** | Hybrid model | **Heavy model** | Light model | Light model | Light model | Light model | Light model | Light model | Light model | **Heavy model** | Light model | Light model | Light model |
| **Publicity available HTA report** | Yes | Yes | Yes | Yes | Yes* | Yes | Yes | Yes | Yes | Yes | Yes | Yes | Yes |

*Ireland – publicity avaiable report only summaries are available.

Source: own elaboration based on systematic review

# **Tab. 6. The scope of HTA - criteria taken into account in the assessment of health technologies**

| **Country** | **Unmet medical need** | **Innovation** | **Clinical efficiency** | **Safety profile** | **Cost effectiveness (profitability)** | **Budget impact (financial consequences, feasibility)** | **Other**  **(ethical, social, legal and organizational aspects)** |
| --- | --- | --- | --- | --- | --- | --- | --- |
| **Poland** | *YES  – not obligatory by law* | *YES*  *– not obligatory by law* | YES  – as a formal criterion | YES  – as a formal criterion | YES  – as a formal criterion | YES  – as a formal criterion | YES  – as a formal criterion |
| **England** | YES  – as a formal criterion | YES  – as a formal criterion | YES  – as a formal criterion | YES  – as a formal criterion | YES  – as a formal criterion | YES  – as of 2017, April | NO |
| **Scotland** | YES  – as a formal criterion | YES  – as a formal criterion | YES  – as a formal criterion | YES  – as a formal criterion | YES  – as a formal criterion | YES  – as a formal criterion | NO |
| **Wales** | *YES  – not obligatory by law* | *YES*  *– not obligatory by law* | YES  – as a formal criterion | YES  – as a formal criterion | YES  – as a formal criterion | YES  – as a formal criterion | NO |
| **Ireland** | *YES  – not obligatory by law* | *YES*  *– not obligatory by law* | YES  – as a formal criterion | YES  – as a formal criterion | YES  – as a formal criterion | YES  – as a formal criterion | NO |
| **France** | YES  – as a formal criterion | YES  – as a formal criterion | YES  – as a formal criterion | YES  – as a formal criterion | NO | NO | NO |
| **the Netherlands** | *YES  – not obligatory by law* | *YES*  *– not obligatory by law* | YES  – as a formal criterion | YES  – as a formal criterion | YES  – as a formal criterion | YES  – as a formal criterion | NO |
| **Germany** | YES  – as a formal criterion | YES  – as a formal criterion | YES  – as a formal criterion | YES  – as a formal criterion | NO | NO | NO |
| **Norway** | *YES*  *– not obligatory by law* | *YES*  *– not obligatory by law* | YES  – as a formal criterion | YES  – as a formal criterion | YES  – as a formal criterion | YES  – as a formal criterion | YES  – as a formal criterion |
| **Sweden** | YES  – as a formal criterion | YES  – as a formal criterion | YES  – as a formal criterion | YES  – as a formal criterion | YES  – as a formal criterion | YES  – not obligatory by law | YES  – as a formal criterion |
| **Canada** | YES  – as a formal criterion | *YES*  *– not obligatory by law* | YES  – as a formal criterion | YES  – as a formal criterion | YES  – as a formal criterion | YES  – as a formal criterion | NO |
| **Australia** | YES  – as a formal criterion | YES  – as a formal criterion | YES  – as a formal criterion | YES  – as a formal criterion | YES  – as a formal criterion | YES  – as a formal criterion | NO |
| **New Zealand** | *YES*  *– not obligatory by law* | *YES*  *– not obligatory by law* | YES  – as a formal criterion | YES  – as a formal criterion | YES  – as a formal criterion | YES  – as a formal criterion | NO |

Source: own elaboration based on systematic review

# **Tab. 7. Analytical methods and techniques in HTA**

| **Categories** | **Poland** | **England** | **Scotland** | **Wales** | **Ireland** | **France** | **the Netherlands** | **Germany** | **Norway** | **Sweden** | **Canada** | **Australia** | **New Zealand** |
| --- | --- | --- | --- | --- | --- | --- | --- | --- | --- | --- | --- | --- | --- |
| **Efficacy** | Mandatory | Mandatory | Mandatory | Mandatory | Mandatory as a supplement to economic analysis | Mandatory | Mandatory | Mandatory | Mandatory | Mandatory | Mandatory | Mandatory | Mandatory |
| **Clinically meaningful oucomes** | Mandatory | Mandatory | Mandatory | Mandatory | Mandatory | Mandatory | Mandatory | Mandatory | Mandatory | Mandatory | Mandatory | Mandatory | Mandatory |
| **Surogate outcomes** | Considered | Considered | Considered | Considered | Considered | Considered | Considered | Considered | Considered | Considered | Considered | Considered | Considered |
| **Quality of life outcomes** | Mandatory | Mandatory | Mandatory | Mandatory | Mandatory | Mandatory | Mandatory | Mandatory | Mandatory | Mandatory | Mandatory | Mandatory | Mandatory |
| **Safety** | Mandatory | Mandatory | Mandatory | Mandatory | Mandatory | Mandatory | Mandatory | Mandatory | Mandatory | Mandatory | Mandatory | Mandatory | Mandatory |
| **Systematic reviews for collecting evidence required** | Mandatory | Mandatory | Mandatory | Mandatory | Mandatory | Mandatory | Mandatory | Mandatory | Mandatory | Not mandatory | Mandatory | Mandatory | Mandatory |
| **Cost efficiency** | Mandatory | Mandatory | Mandatory | Mandatory | Mandatory | Mandatory but separately | Mandatory | Optional | Mandatory | Mandatory | Mandatory | Mandatory | Mandatory |
| **Thresholds (EUR)*** | 3 x GDP per capita for ICUR (QALY) or ICER (LYG)  35 724 EUR | 23 452 EUR -  58 630 EUR | 23 452 EUR -  35 178 EUR | 23 452 EUR - 35 178 EUR | 45 000 EUR | No official threshold | 20 000 EUR -  80 000 EUR | Efficiency frontier | 26 882 EUR | No official threshold (50% likelihood of approval for ICER between 79 400 EUR – 111 700 EUR | No official threshold | No official threshold | No official threshold |
| **Methods** | CUA, CEA – obligatory  CMA – if applicable  CCA | CUA, CEA, CMA | CUA, CEA, CMA | CUA, CEA, CMA | CUA, CEA, CMA | CEA, CUA | CEA, CUA | CBA, CUA, CEA | CUA, CMA | CUA, CEA, CBA | CUA, CEA, CMA | CUA, CEA, CMA | CUA, CEA, CMA |
| **Preferred outcome measure** | QALY or LYG | QALY or LYG | QALY or LYG | QALY or LYG | QALY or LYG | QALY, LYG | QALY or LYG | Patient relevant outcome | QALY or LYG | QALY | QALY or LYG | QALY or LYG | QALY or LYG |
| **Discounting** | Mandatory | Mandatory | Mandatory | Mandatory | Mandatory | Mandatory | Mandatory | Mandatory | Mandatory | Mandatory | Mandatory | Mandatory | Mandatory |
| **Sensivity analysis** | Mandatory | Mandatory | Mandatory | Mandatory | Mandatory | Mandatory | Not mandatory | Mandatory | Mandatory | Mandatory | Mandatory | Mandatory | Mandatory |
| **BIA** | Mandatory | Mandatory | Mandatory | Mandatory | Mandatory | Not mandatory – highly recommended | Mandatory | Mandatory | Mandatory | Not mandatory | Mandatory | Mandatory | Mandatory |
| **Type of costs** | Direct medical, direct non-medical | Direct medical, social | Direct medical, social | Direct medical, social | Direct medical, social | Direct medical, direct non-medical, indirect | Direct, indirect | Direct, informal costs, loss productivity | Direct medical, direct non-medical | Direct medical, direct non-medical, indirect | Direct medical, direct non-medical | Direct medical, direct non-medical | Direct medical, direct non-medical |

Source: own elaboration based on systematic review

# **Tab. 8. Time of reimbursement process**

| **Category** | **Poland** | **England** | **Scotland** | **Wales** | **Ireland** | **France** | **the Netherlands** | **Germany** | **Norway** | **Sweden** | **Canada** | **Australia** | **New Zealand** |
| --- | --- | --- | --- | --- | --- | --- | --- | --- | --- | --- | --- | --- | --- |
| **Duration of HTA agency assessment** | 60-80 days | 245 days (FAD)  305 days (ACD and FAD) | 126 days  154 - 182 days (orphan drugs) | No data | 28 days (initial inspection)  Approx. 90 days (full HTA) | 90 days | No data | 180 days | 156 days | No data | 236 days | No data | No data |
| **Duration of reimbursement decision process by regulation** | 240 days  (drug program)  180 days  (pharmacy  and chemotherapy) | 275 days (FTA)  335 days  (MTA or STA) | No data | No data | 180 days | 180 days | 180 days  68 days - generics | 270 days | 180 days | 180 days | 180 (cancer drugs)  90 (other) | 180 days | No data |
| **Duration of reimbursement process in practice** | 844 days* | 340 days* | 417 days* | No data | 541 days* | 497 days* | 294 days* | 133 days* | 414 days* | 261 days* | 602 days¥ | 467 days** | 789 days*** |

Source: own elaboration based on systematic review

* Average time to reimbursement for innovative treatments (2017-2020) based on EFPIA Patients W.A.I.T. Indicator 2021 Survey Updated July 2022, page 12

¥ Average number of cumulative and segmental days from NOC to at least one provincial listing, by review agency (2012–2016) based on Salek S, Lussier Hoskyn S, Johns JR, Allen N and Sehgal C (2019) Factors Influencing Delays in Patient Access to New Medicines in Canada: A Retrospective Study of Reimbursement Processes in Public Drug Plans. Front. Pharmacol. 10:196. doi: 10.3389/fphar.2019.00196

** Average days from registration to public funding for 143 innovative medicinal products (2011-2020) based on IQVIA analysis Access to Medicines (AtoM 3) November, 2021, page 5

***Average days from registration to public funding for 51 innovative medicinal products (2011-2020) based on IQVIA analysis Access to Medicines (AtoM 3) November, 2021, page 5
